# Knowledge, attitude and practices regarding HIV/AIDS among adult fishermen in coastal areas of Karachi

**DOI:** 10.1186/1471-2458-14-437

**Published:** 2014-05-10

**Authors:** Mubashir Zafar, Nighat Nisar, Masood Kadir, Zafar Fatmi, Zeeshan Ahmed, Kashif Shafique

**Affiliations:** 1 School of Public Health, Dow University of Health Sciences, Karachi, Pakistan; 2 Department of Community Health Sciences, Aga Khan University, Karachi, Pakistan; 3 Institute of Health & Wellbeing, Public Health, University of Glasgow, 1-Lilybank Gardens, Glasgow, United Kingdom

**Keywords:** HIV/AIDS, Pakistan, KAP, Fishermen, Coastal-belt

## Abstract

**Background:**

Migrant populations are at high risk of Human Immuno Deficiency Virus infection (HIV) and Acquired Immunodeficiency Syndrome (AIDS). Studies of HIV/AIDS knowledge, attitudes and practices among fishermen in developing countries have shown gaps in knowledge and fear of contagion with ambivalent attitudes towards HIV/AIDS and inconsistent universal precautions adherence. The aim of this study was to determine the knowledge, attitude and practices regarding HIV/AIDS among adult fishermen in a coastal area of Karachi, Pakistan.

**Methods:**

Community based cross sectional study was conducted among fishermen in coastal area of Karachi from June to September 2012. A total of 297 adult fishermen were selected by using simple random sampling technique from different sectors of coastal village. Data were collected using a structured validated questionnaire. The frequency distribution of both dependent and independent variables were worked out. Comparisons of knowledge, attitude and practices regarding HIV/AIDS by socio-demographic characteristics were made using logistic regression.

**Results:**

Out of 297 fishermen, majority had in-appropriate knowledge (93.6%), negative attitude (75.8%) and less adherent sexual practices (91.6%). In univariate analysis, lower education and higher income were significantly associated (OR 2.25, 95% CI, 1.11, 4.55), (OR = 3.04 CI 1.03-9.02, p value 0.04) with negative attitude and un-safe practices towards HIV/AIDS respectively, whereas no significant association of socio-economic characteristics with knowledge, attitude and practices were observed in multivariate analysis.

**Conclusions:**

This study suggests that fishermen had very poor knowledge, negative attitudes towards HIV and AIDS and had unsafe sexual practices which suggest that they lack the basic understanding of HIV/AIDS infection. Extensive health education campaign should be provided to the vulnerable sections of the society for the control of HIV/AIDS.

## Background

HIV/AIDS is a major health problem, especially for the developing world. The World Health Organization (WHO) reported approximately 33.3 million people were infected with HIV/AIDS globally in the year 2009 [1]. More than seven thousands new HIV/AIDS infections occur every day while approximately 4000 people die every day globally with HIV/AIDS [1]. Of those who are infected, nearly 95% were unaware of their HIV/AIDS status [2-4]. Due to sexual transmission of HIV/AIDS certain groups are at higher risk of contracting the disease including commercial sex workers, Intra Venous (IV) drug abusers and mobile population like military personnel, fishermen, prisoners and truck drivers [5,6]. Fishermen have been reported at highest risk [6] because they have social linkages and they move to various sites, and regional markets. HIV/AIDS prevalence among fishermen is reported to be 4–14 times higher than the general population [5-10].

Evidence suggests that the basic knowledge of HIV among fishermen is very low. In a KAP survey in Indian state of Kerala, only 5% of the fishermen reported that they know about HIV and AIDS [11]. Some other studies indicate that about half of the fishermen knew about modes of transmission of HIV, 44.9% attribute to positive living and only 39.6% had knowledge about preventive services for HIV/AIDS [7,10-12].

Fishermen are considered a high risk bridging population for HIV/AIDS because of their social contacts and interactions with general population. The key components of that bridging phenomenon are they stay away from home for long periods and most people involved in fishing as an occupation are within the age groups (18 to 35 years) most vulnerable to sexually transmitted infections (STIs). As they stay away from home for longer durations, the chances of indulging in risky behavior such as unsafe sex with casual/commercial sex worker substantially increases. Studies suggest that frequent mobility and high levels of alcohol use among fishermen before and during sexual encounters may be a factor of unsafe sexual practices [5,13].

Although regional evidence reflects that fishermen are a high risk population group, however, knowledge, attitudes and practices of this population group in Pakistan, largely remains unknown. Given that fairly large population groups are associated with fishing business, assessment of fishermen knowledge, attitudes and practices is important to understand their needs. Therefore, the objective of this study was to determine level of knowledge, attitude and practices regarding HIV/AIDS among adult fishermen in a coastal area of Karachi.

## Methods

### Study area, design and period

This cross-sectional study was conducted among fishermen living in coastal areas of Karachi from June to September, 2012. During the study period there were approximately 60,000 individuals living in the coastal village of Bin Qasim town, Karachi.

### Sample size and sample technique

Sample was calculated using WHO software for sample size determination in health studies; sample size was calculated based on proportion of knowledge, attitude and practices regarding of HIV/AIDS in fishermen reported by a previous study [11]. The sample size was estimated by using proportions of having appropriate knowledge, positive attitude and safe practices as 5%, 7.5% and 15% respectively at confidence level 95% and bound of error 3%, the estimated sample size came out to be 203, 245 and 297 respectively. Therefore, taking the largest sample, a minimum of 297 fishermen were included in the study. The sampling technique employed was a simple random sampling, using the list of all fishermen obtained from the fisher folk society which is the representative organization of fishermen. The list is upgraded every year and the most recent list was used at the time of data collection as a sampling frame.

### Instrument and data collection

Fishermen were defined as all individuals living in fishing communities and involved in or depending on fishing for economic survival. These individuals have high risk for transmitting infection because they can carry and transmit infection to their spouses and sexual partners. They also move to general population where they have contacts and can transmit infection [9]. All fishermen of age 18 to 60 years and working for at least 2 years were included in the study. The study instrument was a validated questionnaire [14] which comprised of four sections. Part 1 was related to the sociodemographic characteristics, part 2, 3 and 4 related to knowledge, attitude and practices of fishermen regarding HIV/AIDS respectively. Knowledge of HIV/AIDS of fishermen was assessed by 21 items questionnaire which included questions regarding transmission of HIV/AIDS, sign, symptoms and preventive measure. Attitude towards HIV/AIDS were assessed by a 16-items questionnaire which included questions on shaking hands, eating in same utensils, sharing room, working with HIV/AIDS patient. Practices were assessed by a 21-items questionnaire which included use of condom during sexual act, multiple sexual partners, HIV testing, treatment of STI.

The questionnaire was prepared in English version and translated to the local language (Sindhi) and checked for consistency. Before the final data collection, this questionnaire was pre-tested on fifty fishermen in another coastal village of Karachi and the results were used to improve the phrasing of questions in this instrument.

### Scoring

The knowledge scale was based on an instrument developed by Eckstein [15] containing statements about disease presentation, transmission, precaution and prevention. Each item was scored as ‘yes’, ‘no’ or ‘don’t Know’ with 2, 0 and 1 scores respectively. Correct responses were summed on a 38-point rating scale and those who had a score of HIV/AIDS knowledge index equal or more than 23 considered as having appropriate HIV/AIDS knowledge and those who had scores below 23 were considered as having inappropriate knowledge. The attitude scale is based on an instrument for measuring attitudes towards HIV/AIDS [16], and comprised of items probing empathic and avoidance behavior. Likert scales ranging from disagreement; do not have any idea and agreement, with 0, 1 and 2 scores accordingly. Correct responses were summed on 16- point rating scale and those who had attitude toward HIV/AIDS index equal to 10 or more considered as positive HIV/AIDS attitude whereas those who had score below 10 were considered as negative attitudes. The practices scale [17] consisted of questions relating to universal precautions adherence, post-exposure prophylaxis (PEP) requirement and behavior with regard to HIV testing and referral. Items were scored as ‘Yes’ , ‘No’ or ‘not applicable’ with 2, 0 and 1 scores respectively. Correct responses were summed up to 38 point rating scale and those who had safe practice in relation to HIV/AIDS equal to or more than 23 were considered more adhere to safe practice and those who scored below 23 were considered as less adhere to safe practice. Three variables which were dichotomous i.e. Knowledge (Appropriate/In-Appropriate) Attitude (Positive/Negative) and Practice (Safe/Un-Safe) were used as outcome variables. These above mentioned scales have been used in many countries such as USA, Sweden and countries in Africa, in different studies [18-20] regarding the knowledge, attitude and practices assessment about the HIV/AIDS of the population.

### Data management and analysis

During data collection process, the data were checked for completeness and any incomplete or misfiled questions were sent back for correction. Data were double entered in Epi Data software version 1.3, cleaned for missing values and checked error rate which was 0.02%. Data were analyzed using software SPSS version 16. Descriptive statistics were used to describe socio-demographic characteristics. The frequency distribution of both dependent and independent variables were worked out. Logistic regression models were used to examine the possible association between independent variables and outcome variables. A p-value ≤ 0.05 was considered significant. Independent variables which were biologically plausible to have some confounding potential or known factors associated with KAP or those appeared with a p-value ≤0.25 in unadjusted analysis were included in the multivariate analysis.

### Ethical consideration

The study was ethically approved by the Ethical Review Committee of Aga Khan University Karachi. Written consent, after explanation about the study, was obtained from the study participants. The interviews were conducted in a private room for reasons of privacy, confidentiality of information. Counseling was done after interview providing them basic information, positive attitudes and safe practices which can prevent the spread of HIV/AIDS. Those fishermen who had negative attitude and un-safe practices were referred to tertiary care hospitals for screening of the disease. Study participants had rights to withdraw from the study at any time.

## Results

Mean age of fishermen was 32 ± 12.1 years, 60.9% were of age 18–30 years, 80.8% were married, 74.4% were illiterate and 63.6% were working on daily wages. 65.3% were earning less than 10,000 Pakistani rupees and 66.2% of the fishermen were not able to save money from their earned income.

Mean scores of knowledge, attitude and practice about HIV/AIDS of fishermen were 14.17 (SD = 5.29), 11.96(SD = 9.34), 14.47(SD = 5.25) and median scores were 13 (IQR =11-18), 10 (IQR = 3–18) and 13 (IQR = 3–28) respectively. Majority of fishermen had inappropriate knowledge (93.6%) of HIV/AIDS, 75.8% had negative attitude towards HIV/AIDS and 91.6% were involved in unsafe sexual practices.

The major sources of information regarding HIV/AIDS were electronic media (TV, radio) (n = 101, 34%), followed by print media (Newspaper) (n = 14, 4.7%). 89.6% of the fishermen were addicted of substances like nicotine, niswar and gutka (Table 1).

Majority of fishermen (61.6%) did not hear about HIV/AIDS and 38% believed that even healthy looking individual could transmit infection (Table 2). 50.5% knew that HIV/AIDS could be transmitted through un-protected sexual contact and un-safe blood transfusion. 44.4% of respondents had known that HIV can be transmitted through needle stick injury while 24.6% knew that it can be transmit through placenta. 86.9% believed that HIV/AIDS is incurable whereas 67.3% and 60.6% disagreed on living and working with HIV/AIDS patients respectively.

**Table 1:** Baseline sociodemographic characteristics of fishermen participated in KAP (knowledge, attitude and practices) survey

**Variables**	**Frequency (n)**	**Percentage (%)**
**Age (years) Mean (SD)** 32 (12.1)		
18-30	181	60.9
31-60	116	39.1
**Marital status**		
Single	57	19.2
Married	240	80.8
**Educational status**		
Illiterate^*^	221	74.4
Literate	76	25.6
**Employment status**		
Daily wages	189	63.6
Contracted	108	36.4
**Income level**		
< 10000**	194	65.3
≥ 10000	103	34.7
**Saving from income**		
Yes	100	33.8
No	197	66.2
**Addiction from any substance**		
Yes	266	89.6
No	31	10.4
**Type of addiction of substance**		
Smokeless tobacco^†^	248	93.2
Smoking cigarette	18	6.8
**Frequency of smokeless tobacco**		
< 10 packets/day	27	10.9
≥10 packets/day	221	89.1
**Frequency of smoke tobacco**		
< 10 cigarettes/day	6	33.3
≥10 cigarettes/day	12	66.7
**Sources of HIV/AIDS information**^**¶**^		
Print media (news paper)	14	4.7
Electronic media (tv, radio)	101	34.0

^*^Cannot read and write, ^**^Pakistani Rupee, ^†^Gutka, Pan, Areca ^¶^values not equal to 100%.

**Table 2:** Knowledge, attitude and practices regarding HIV/AIDS

**Knowledge, attitude and practices regarding HIV/AIDS**	**Correct response**	**Frequency (n)**	**Percentage (%)**
Heard about HIV/AIDS	True	114	38.4
Healthy looking individual having HIV/AIDS	True	113	38.0
HIV/AIDS curable disease	True	42	14.1
HIV/AIDS transmits by un protected sexual contact	True	150	50.5
HIV/AIDS transmits by unsafe blood transfusion	True	140	47.1
HIV/AIDS transmits by needle stick injury	True	132	44.4
HIV/AIDS transmits from mother to child during pregnancy	True	73	24.6
Agree to live with HIV/AIDS person	Agree	97	32.7
Agree to work with HIV/AIDS person	Agree	102	39.3
Agree to provide social support to HIV/AIDS person	Agree	117	39.4
Agree to send their children in same school with children of HIV/AIDS	Agree	85	28.6
Circumcision practices	Yes	288	97.0
More than one sexual partner (Spouse)	No	29	9.8
Condom usage	Yes	76	25.6
Sexual partner other than spouse	No	76	25.6
STI informed to sexual partner	Yes	14	4.7
Received treatment for STI	Yes	31	10.4

Circumcision practices (97.0%), more than one spouse as sexual partner (9.8%), condom usage in last one year (25.6%), sexual partners other than spouse (25.6%) were prevalent characteristics among fishermen. Only a small percentage was (4.7%) informed about STI and of these only, 10.4% received treatment for STI.

Illiterate fishermen were two (OR = 2.25, CI 1.11- 4.55, p value 0.02) times more likely to have negative attitude towards HIV/AIDS than literate while fishermen who earned ≥10000 rupees per month were three (OR = 3.04 CI 1.03-9.02, p value 0.04) times more likely to have unsafe practices than who earned less. No significant association was observed for in-appropriate knowledge and other socio-demographic variables (Table 3).

**Table 3:** Comparison of knowledge, attitude and practices levels’ regarding HIV by sociodemographic characteristics(un-adjusted)

**Variables**	**Knowledge**			**Attitude**			**Practices**		
	**In-appropriate knowledge**	**Un-adjusted OR (95% CI)**	**P- value**	**Negative attitude**	**Un-adjusted OR (95% CI)**	**P- value**	**Unsafe practices**	**Un-adjusted OR (95% CI)**	**P- value**
**Age (years)**									
< 30	172	0.55 (0.21-1.40)	0.21	134	1.27 (0.73-2.22)	0.38	168	0.67 (0.29-1.52)	0.34
≥ 30	106	1		91	1		104	1	
**Marital status**									
Single	52	1.55 (0.53-4.50)	0.41	44	0.90 (0.45-1.79)	0.77	218	0.55 (0.15-1.90)	0.34
Married	226	1		181	1		54	1	
**Education status**									
Illiterate^*^	206	1.31 (0.42-4.07)	0.64	160	2.25 (1.11-4.55)	0.02	200	1.89 (0.62-5.69)	0.25
Literate	72	1		65	1		72		
**Fishing activities**									
≥ 2 weeks	29	0.99 (0.21-4.50)	0.99	200	1.37 (0.54-3.49)	0.50	242	2.97 (0.38-22.79)	0.29
< 2 weeks	249	1		25	1		30	1	
**Income level**									
≥ 10000^**^	94	0.56 (0.22-1.44)	0.23	143	1.39 (0.78-2.47)	0.26	173	3.04 (1.03-9.00)	0.04
< 10000	184	1		82	1		99	1	
**Tobacco user**									
Smokeless tobacco^†^	231	0.93 (0.20-4.26)	0.93	185	1.70 (0.47-6.07)	0.41	225	0.67 (0.15-3.01)	0.60
Smoking cigarette	18	1		15	1		18	1	

^*^Cannot read and write, ^**^Pakistani Rupee, ^†^Gutka, Pan, Areca.

p-value was calculated by logistic regression and significant at the ≤0.05 level.

On multivariate analysis, men who were singles, had monthly income ≥ 10,000 were more likely to have in-appropriate knowledge, while those who were illiterate were more likely to have negative attitudes and un-safe practices compared to literate individuals, however, these associations were not statistically significant after adjusting for co-variates. There were no significant associations of socio-demographic characteristics with level of knowledge, attitude and practices towards HIV/AIDS after adjusting for co-variates (Table 4).

**Table 4:** Comparison of knowledge, attitude and practices levels’ regarding HIV by sociodemographic characteristics (adjusted)

**Variables**	**Knowledge**			**Attitude**			**Practices**		
	**In-appropriate knowledge**	**AOR**^**†† **^**(95% CI)**	**P- value**	**Negative attitude**	**AOR (95% CI)**	**P- value**	**Unsafe Practices**	**AOR (95% CI)**	**P- value**
**Age (years)**									
< 30	172	0.39 (0.11-1.38)	0.14	134	1.25 (0.66-2.39)	0.48	168	0.71 (0.27-1.84)	0.48
≥ 30	106	1		91	1		104	1	
**Marital status**									
Single	52	2.39 (0.60-9.46)	0.21	44	0.72 (0.33-1.57)	0.42	218	0.65 (0.17-2.53)	0.54
Married	226	1		181	1		54	1	
**Education status**									
Illiterate^*^	206	1.29 (0.34-4.84)	0.69	160	1.90 (0.86-4.20)	0.11	200	1.60 (0.43-5.83)	0.47
Literate	72	1		65	1		72		
**Fishing activities**									
≥ 2 weeks	29	0.52 (0.06-4.29)	0.54	200	0.38 (0.11-1.36)	0.14	242	0.51 (0.06-4.10)	0.52
< 2 weeks	249	1		25	1		30	1	
**Income level**									
≥ 10000^**^	94	1.89 (0.67-5.28)	0.22	143	0.80 (0.42-1.54)	0.51	173	0.41 (0.13-1.29)	0.12
< 10000	184	1		82	1		99	1	
**Tobacco user**									
Smokeless tobacco^†^	231	0.90 (0.10-3.26)	0.95	185	1.47 (0.40-5.39)	0.56	225	0.92 (0.25-2.01)	0.40
Smoking cigarette	18	1		15	1		18	1	

^*^Cannot read and write, ^**^Pakistani Rupee, ^†^Gutka, Pan, Areca. ^††^Adjusted Odd ratio.

p-value was calculated by logistic regression and significant at the ≤0.05 level.

Adjusted for age, marital status, educational status, fishing activities, income level, tobacco use.

## Discussion

This survey revealed that considerable proportion of fishermen have in-appropriate knowledge, negative attitude and un-safe practice regarding HIV/AIDS and also identified several deficiencies in the knowledge, attitude and practices of HIV/AIDS in certain key areas, such as basic knowledge about the disease, transmission and ways to prevent it.

These results are in concordance with the level of HIV/AIDS knowledge found in other studies from developing countries like African and Asian pacific region [11-13], suggesting that knowledge among fishermen is very low which may perhaps be due to high level of illiteracy (Most of fishermen in this study reported that more than two third of participants were illiterate) and they have not heard about HIV/AIDS. This is in contrast to the findings of Pakistan Demographic Health Survey (PDHS 2006–2007) where, more than two third of population had heard about HIV/AIDS [21]. In PDHS, general population literacy rate was around 40%. When they were asked about mode of spread and preventive strategies for HIV/AIDS, a high percentage did not know about the source through which the infection could spread, which is also quite dissimilar to the findings reported in a national survey of Pakistan [22], this may be because national survey represents the general population where literacy rate was high and they had more access to HIV information. Most of the participants believe that HIV/AIDS prevent by less sexual activity/ faithful to sexual partner, which is consistent with results of PDHS 2006–2007 [21] and studies from other regions of the world including African countries [23-25].

In this study there was no significant difference of knowledge among different socio-demographic groups. This is perhaps due to the fact that the knowledge about HIV/AIDS, in general, is fairly poor across all study participants. This is in contrast to some others studies where education level, age categories were significantly associated with HIV/AIDS knowledge level among fishing communities [11,12,23]. One reason for the low level of sexual knowledge reported in these studies is that, as a conservative Muslim society, Pakistan has certain social and cultural barriers to discuss and address the problems pertaining to sexuality or STD, including HIV/AIDS. In this study major source of information of HIV/AIDS were electronic media, but fishermen were out of home for fishing purpose and HIV information was not accessible to them through electronic media [26].

Generally participants had negative attitude towards HIV/AIDS but they were sympathetic towards HIV/AIDS patient and against isolating the patient form society. Other investigators have reported consistent presentation for negative attitude towards HIV/AIDS among fishermen [11,12]. Social and culture attitude play an important role-play in the perception and response to danger. Study found that fishermen had no perception of such type of danger of HIV/AIDS and believes that everything come from nature and solved with time [27]. The denial of danger and fatalism are common themes among fishermen [27]. In this study only significant difference of attitude was observed among those who were literate compared with illiterates This is in line with the findings of other studies [28,29].

No participant reported that he has gone through screening test of HIV/AIDS and this is due to unawareness about the disease, access and availability of such services [30]. The Condoms uses among study participants were very low (25.6%) which is consistent with the findings of other studies [24,31]. The reason for not using condoms because they have no access to information and social marketing companies are not allowed to work in this particular area due to culture barriers. In this study, one quarter of the men reported extra-marital sex, this sexual practice could be high and may be underreported, therefore it should not be ignored as a potential risk factor while another study had reported that nearly half of men had extra-marital sex [32]. Men with higher income appeared to have unsafe sexual practices in this study, this may be perhaps reflecting that those who have higher income more likely to contact paid-sex workers [33-35].

There are several strength of this study, finding of this study determine the high risk group which was previously ignored, fishermen may act as a bridging population because they have social contact with the general population which may lead to transfer of infection from high risk group to low risk group (general population). Fishery plays an important role in Pakistan's economy, during the year 2000, Rs.7.9 billion valued of fish and fishery products were exported [36]. Loss of human resources will jeopardize the economy of Pakistan. The findings of this study should be considered in light of the following limitations. We relied on self-report to assess the sensitive health risk behaviors. Due to social and cultural reasons, participants may have hidden some risk behaviours, our measures of association may be an underestimate of the true effect. We couldn’t observe any statistically significant differences in knowledge, attitude and practice scores, this may be because majority of participants had poorer knowledge, negative attitudes and unsafe practices related to HIV and AIDS, so the numbers in high knowledge, positive attitude and safe practice groups were smaller to observe any convincing associations. A larger cross-sectional survey, using multiple cities and centers may provide further insights and identify some high-risk groups. Finally, our study did not cover distribution of multiple sexual relationships, mobility, and port based IDU among fishermen, which are the risk factors of HIV among fishermen, so this remains a limitation of the study.

Despite these limitations, appropriate health education should be given to the fishermen in a generalized way to bring behavioral change targeting at all health-risk behaviors in relation to HIV and AIDS. Electronic media appeared to be the only source which was utilized by at least one third of the populations, so behavior change interventions may be executed through local radio/TV channels to improve the knowledge and practices.

## Conclusion

Study identified substantial gap in the knowledge, negative attitude towards HIV/AIDS and un-safe sexual practice among fishermen in Rehri Goth, coastal area of Karachi. Mass education related to HIV/AIDS among the fishermen is urgently needed.

## Competing interests

The authors declare that they have no competing interests.

## Authors’ contributions

MZ conceived the study, undertook statistical analysis and drafted the manuscript. NN, MK, ZF, KS supervised the study and made major contributions to the study design and statistical analysis. ZA contributed to the writing of the manuscript and all authors approved the submitted version of the manuscript.

## Pre-publication history

The pre-publication history for this paper can be accessed here:

http://www.biomedcentral.com/1471-2458/14/437/prepub

## References

[B1] UNAIDS Global Report: UNAIDS Report on the Global AIDS Epidemic 2012 Geneva: UNAIDS Available at http://www.unaids.org/en/media/unaids/contentassets/documents/epidemiology/2013/gr2013/UNAIDS_Global_Report_2013_en.pdf. Website

[B2] Excler JL Parks CL Ackland J Rees H Gust ID Koff WC Replicating viral vectors as HIV vaccines Biologicals 2009 38 4 511 521 20537552 10.1016/j.biologicals.2010.03.005

[B3] Nico J Arora P Jha P Williams B Mackinon L Vlas S The rise and fall of HIV in high-prevalence countries: a challenge for mathematical modeling PLoS Comput Biol 2014 10 3 30459 10.1371/journal.pcbi.1003459 PMC3952813 24626088

[B4] David W Brian A Blair W Jane M A systematic review of the frequency and correlates of partner abuse in HIV-infected women and Men Who partner with Men J Assoc Nurses AIDS Care 2014 25 1 15 35 10.1016/j.jana.2013.04.003 PMC3875616 24070646

[B5] FAO Food and Agricultural Organization of the United Nations 2006 Impact of HIV/AIDS on Fishing Communities: FAO [Progress report]. Available at http://www.fao.org/hivaids/publications/hivaids.pdf. Website

[B6] FAO Global Report: Food and Agricultural Organization of the United Nations 2006 HIV and AIDS in fishing communities: public health issue but also fisheries development and management concern. The State of World Fisheries and Aquaculture Available at http://www.fao.org/newsroom/common/ecg/1000544/en/hivfishingEN.pdf. Website

[B7] Kissling E Allison EH Seeley JA Russell S Bachmann M Musgrave SD Heck S Fisherfolk are among groups most at risk of HIV: cross-country analysis of prevalence and numbers infected AIDS 2005 19 17 1939 10.1097/01.aids.0000191925.54679.94 16260899

[B8] Allison EH Seeley JA HIV and AIDS among Fisherfolk: a threat to ‘responsible fisheries’? Fish Fisheries 2004 5 3 215 234 10.1111/j.1467-2679.2004.00153.x

[B9] Nagoli J Holvoet K Remme M HIV and AIDS vulnerability in fishing communities in Mangochi district, Malawi Afr J AIDS Res 2010 9 1 71 80 10.2989/16085906.2010.484575 25860415

[B10] Olowosegun T Sule AM Sanni OA Onimisi HU Olowosegun OM Awareness of HIV/AIDS pandemic in selected fishing communities in North Central Nigeria Asian J Epidemiol 2008 1 1 17 23 10.3923/aje.2008.17.23

[B11] George M KAP Study on HIV/AIDS Among Fishermen Community in a Coastal Area of Kerala 2008 Thiruvananthapuram: National conference on students medical research medical College Available at http://communitymedicinetvm.org/demo/undergraduate-research-papers/. Website

[B12] Sheikh NS Sheikh AS Awareness of HIV and AIDS among fishermen in coastal areas of Baluchistan J Coll Phys Surg Pak 2003 13 4 192 12718771

[B13] Setiawan IM Patten JH The organization of STI/HIV risk-taking among long-line fishermen in Bali, Indonesia Int Marit Health 2010 62 4 201 21348013

[B14] UNAIDS HIV/AIDS Prevention Indicator Survey, Knowledge, Attitude and Sexual Behavior 2004 UNAIDS Available at http://www.globalhivmeinfo.org/DigitalLibrary/Digital%20Library/HIV%20AIDS%20Prevention%20Indiciator%20Survey.pdf. Website

[B15] Eckstein EC Knowledge and Attitude of Nurses Regarding Patient With Acquired Immunodeficiency Syndrome Thesis presented to the Faculty of the Frances Payne Bolton School of Nursing 1987 Cleveland, OH, USA: western Reserve University Available at http://onlinelibrary.wiley.com/doi/10.1111/j.1744-6198.1984.tb01109.x/abstract. Website

[B16] Rubin KH Burgess KB Dwyer KM Hastings PD Predicting preschoolers’ externalizing behaviors from toddler temperament, conflict, and maternal negativity Develop Psycho 2003 39 1 164 12518817

[B17] Delobelle P Rawlinson JL Ntuli S Malatsi I Decock R Depoorter AM HIV/AIDS knowledge, attitudes, practices and perceptions of rural nurses in South Africa J Adv Nurs 2009 65 5 1061 1073 10.1111/j.1365-2648.2009.04973.x 19399982

[B18] Rosenberg P Biggar R Trends in HIV incidence among young adults in the united states JAMA 1998 279 23 1894 1899 10.1001/jama.279.23.1894 9634261

[B19] Kohi TW Horrocks MJ The knowledge, attitudes and perceived support of Tanzanian nurses when caring for patients with AIDS Int J Nurs Stu 1994 31 1 77 86 10.1016/0020-7489(94)90009-4 8194938

[B20] Adelekan ML Jolayemi SO Ndom RJ Adegboye J Babatunde S Tunde M Yusuff O Makanjuola AB Caring for people with AIDS in a Nigerian teaching hospital: staff attitudes and knowledge AIDS Care 1995 7 1 63 72 10.1080/09540129550126849 7632786

[B21] National institute of Population studies of Pakistan Pakistan Demographic Health Survey 2006–07 2007 [Report] Available at http://www.nips.org.pk. Website

[B22] National institute of population studies of Pakistan Reproductive Health of Youth: Perceptions, Attitudes and Practices 2003 [Report]; Available at http://www.nips.org.pk. Website

[B23] Ismail S Hgiogis F Legsse D Alemu E Regassa K Abdella M Shibeshi M Knowledge, attitude and practice on high risk factors pertaining to HIV/AIDS in a rural community Ethiop Med J 1995 33 1 6 7895741

[B24] Smit R HIV/AIDS and the workplace: perceptions of nurses in a public hospital in South Africa J Adv Nurs 2005 51 1 22 29 10.1111/j.1365-2648.2005.03456.x 15941457

[B25] Allison EH Linking National Fisheries Policy to Livelihoods on the Shores of Lake Kyoga, Uganda 2003 Norwich UK: Working Paper No9, Overseas Development Group, University of East Anglia Available at http://www.uea.ac.uk/dev/odg/ladder/. Website

[B26] Spring AO Globalization from below: social movements and altermundism reconceptualizing security from a Latin american perspective Globaliz Environ Challeng 2008 3 379 402 10.1007/978-3-540-75977-5_26

[B27] Poggie J Pollnac RB Jones S Perceptions of vessels safety regulations: a southern New England fishery Mar Policy 1995 19 411 418 10.1016/0308-597X(95)00015-X

[B28] Pickering H Okongo M Ojwiya A Yirrell D Whitworth J Sexual networks in Uganda: mixing patterns between a trading town, Its rural hinter land and a nearby fishing village Int J STD AIDS 1997 8 495 500 10.1258/0956462971920640 9259497

[B29] Seeley JA Malamba SS Nunn AJ Mulder DW Kengeya JF Barton TG Socio-economics tatus, gender and risk of HIV-1 infection in a rural community in South West Uganda Med Anthropol Q 1994 8 78 89 10.1525/maq.1994.8.1.02a00060

[B30] Chua P Harthorn BH, Oaks L Governing migrants sexual behavior work HIV/AIDS and condom use campaigns in Southeast Asia Risk, Culture, and Health Inequality: Shifting Perceptions of Danger and Blame 2005 McGraw-Hill: Westport, Connecticut 165 184

[B31] USAID Global Report 2000 Cambodia: Sexual behavior, STI, and HIV among men who have sex with men in Phnom Penh Available at http://www.ternyata.org/wpcontent/uploads/2010/02/MSM_in_Phnom_Penh_full_report1.pdf. Website

[B32] Nunn A Wagner U Okongo M Malamba S Kengeya JF Mulder D HIV-1 infection in a Ugandan town on the Trans Africa highway prevalence and risk factors J STD AIDS 1996 7 123 130 10.1258/0956462961917320 8737337

[B33] Percin F Akyol O Davas A Saygi H Occupational health of Turkish Aegean small-scale fishermen Occup Med 2012 62 2 148 151 10.1093/occmed/kqr181 22113895

[B34] Zelnick J Odonnell M The impact of the HIV/AIDS epidemic on fishing community in Kwa Zulu Natal, South Africa: nurses’ perspectives and implications for health policy J Public Health Policy 2005 26 2 163 185 10.1057/palgrave.jphp.3200021 16022210

[B35] Khan MW Country Review: Pakistan Review of the State of World Marine Capture Fisheries Management 2006 World Marine Capture Fisheries Management: Indian Ocean Available at http://books.google.com.pk/books?hl=en&lr=&id=_7JD1V3PijUC&oi=fnd&pg=PA281&dq=+Khan+MW.+Country+Review:+Pakistan+Review+of+the+state+of+world+marine+capture+fisheries+m. Webcite

[B36] Khan OA Hyder AA Responses to an emerging threat: HIV/AIDS policy in Pakistan Health Policy Plan 2001 16 2 214 10.1093/heapol/16.2.214 11358924

